# Leaving no‐One Behind: Evidence on the SDGs From the Campbell Collaboration

**DOI:** 10.1002/cl2.70043

**Published:** 2025-04-08

**Authors:** Amanda Newell, Vivian Welch

**Affiliations:** ^1^ Bruyère Research Institute Ottawa Ontario Canada; ^2^ Campbell Collaboration Philadelphia USA

On September 25, 2015, all United Nations member states adopted a shared vision for 2030:

We envisage a world free of poverty, hunger, disease and want, where all life can thrive. We envisage a world free of fear and violence. A world with universal literacy. A world with equitable and universal access to quality education at all levels, to health care and social protection, where physical, mental and social well‐being are assured. A world where we reaffirm our commitments regarding the human right to safe drinking water and sanitation and where there is improved hygiene; and where food is sufficient, safe, affordable and nutritious. A world where human habitats are safe, resilient and sustainable and where there is universal access to affordable, reliable and sustainable energy.

(United Nations General Assembly 2015, 3).

Since then, governments, non‐governmental organizations and countless other stakeholders have multilaterally committed to this vision, adopted as the Sustainable Development Goals (SDGs). With an ambitious plan and progress slowed or halted in several areas by ongoing global challenges, now is the time to reconvene and make new strides (United Nations General Assembly Economic and Social Council 2024). If we hope to achieve transformative progress towards the SDGs over these next 5 years, there must be sufficient evidence to support our actions. The Campbell Collaboration has committed to providing this evidence by publishing systematic reviews and evidence‐gap maps that advance the SDGs in our *2023–2025 strategy* (https://www.campbellcollaboration.org/wp-content/uploads/2024/10/Campbell-strategy-2023-2025-public-draft.pdf).

The virtual issue that follows provides crucial evidence for decision‐makers in SDG progress areas, specifically climate action, gender equality, peace and justice, clean water and sanitation, no poverty, zero hunger, reduced inequalities, good health and well‐being, decent work and economic growth, quality education, and sustainable cities and communities. In doing so, we hope to contribute to a world where no one is left behind.

This collection aims to uphold *SDG 10: Reduced Inequalities* by exemplifying the diversity of our author teams, including teams from India, China, Canada, the United Kingdom, the Netherlands, Belgium, Italy, Sweden, Argentina, and Kenya, and established scholars as well as early career researchers and graduate trainees. Over half of these reviews were funded by national research funding bodies or evidence intermediaries. We welcome proposals for evidence synthesis and methodological research, as well as new editors and peer referees. Our growing early career research network aims to involve evidence‐synthesis researchers from all backgrounds. Get in touch if you are interested! info@campbellcollaboration.org.
